# Electronic Properties of Group-III Nitride Semiconductors and Device Structures Probed by THz Optical Hall Effect

**DOI:** 10.3390/ma17133343

**Published:** 2024-07-05

**Authors:** Nerijus Armakavicius, Philipp Kühne, Alexis Papamichail, Hengfang Zhang, Sean Knight, Axel Persson, Vallery Stanishev, Jr-Tai Chen, Plamen Paskov, Mathias Schubert, Vanya Darakchieva

**Affiliations:** 1 Center for III-Nitride Technology (C3NiT-Janzén), Linköping University, 581 83 Linköping, Sweden; philipp.kuhne@liu.se (P.K.); alexis.papamichail@liu.se (A.P.); vallery.stanishev@liu.se (V.S.); plamen.paskov@liu.se (P.P.); mschubert4@unl.edu (M.S.); 2 Department of Physics, Chemistry and Biology (IFM), Linköping University, 581 83 Linköping, Sweden; 3 SweGaN AB, 582 78 Linköping, Sweden; 4 Department of Electrical and Computer Engineering, University of Nebraska-Lincoln, Lincoln, NB 68588, USA; 5 NanoLund, Center for III-Nitride Technology (C3NiT-Janzén), Terahertz Materials Analysis Center, THeMAC and Solid State Physics Division, Lund University, 221 00 Lund, Sweden

**Keywords:** electrical properties, free charge carriers, optical Hall effect, terahertz, group-III nitrides, gallium nitride, aluminum nitride, aluminum gallium nitride, high-electron-mobility transistor, HEMT

## Abstract

Group-III nitrides have transformed solid-state lighting and are strategically positioned to revolutionize high-power and high-frequency electronics. To drive this development forward, a deep understanding of fundamental material properties, such as charge carrier behavior, is essential and can also unveil new and unforeseen applications. This underscores the necessity for novel characterization tools to study group-III nitride materials and devices. The optical Hall effect (OHE) emerges as a contactless method for exploring the transport and electronic properties of semiconductor materials, simultaneously offering insights into their dielectric function. This non-destructive technique employs spectroscopic ellipsometry at long wavelengths in the presence of a magnetic field and provides quantitative information on the charge carrier density, sign, mobility, and effective mass of individual layers in multilayer structures and bulk materials. In this paper, we explore the use of terahertz (THz) OHE to study the charge carrier properties in group-III nitride heterostructures and bulk material. Examples include graded AlGaN channel high-electron-mobility transistor (HEMT) structures for high-linearity devices, highlighting the different grading profiles and their impact on the two-dimensional electron gas (2DEG) properties. Next, we demonstrate the sensitivity of the THz OHE to distinguish the 2DEG anisotropic mobility parameters in N-polar GaN/AlGaN HEMTs and show that this anisotropy is induced by the step-like surface morphology. Finally, we present the temperature-dependent results on the charge carrier properties of 2DEG and bulk electrons in GaN with a focus on the effective mass parameter and review the effective mass parameters reported in the literature. These studies showcase the capabilities of the THz OHE for advancing the understanding and development of group-III materials and devices.

## 1. Introduction

The advent of group-III nitride semiconductor materials has revolutionized solid-state lighting [1,2] and radio frequency (RF) wireless communication [3,4] and it is already advancing power electronics in a critical and impactful way [5,6]. Indium nitride (InN), gallium nitride (GaN), and aluminum nitride (AlN) compounds and their alloys are direct bandgap semiconductors with bandgap energies ranging from 0.7 to 6.0 eV, facilitating their utilization in optoelectronics and photonics [7]. The GaN-based blue light-emitting diodes (LEDs) have enabled efficient and energy-saving lighting, for which the Nobel Prize in Physics 2014 was awarded. GaN and AlN have high critical electric fields, high saturation carrier velocities, and high thermal conductivity parameters, which make them promising candidates for replacing silicon (Si) in next-generation power electronic devices. The polarization-induced two-dimensional electron gas (2DEG), formed at the interface of AlGaN and GaN has enabled GaN-based high-electron-mobility transistors (HEMTs) [3,8]. These devices are suitable for high-power switching, power amplification, and high-frequency applications in the millimeter-wave range such as next-generation 5G and 6G communication systems, radars, and satellites [4].

Understanding the fundamental material parameters unambiguously drives the development of device technology forward. Especially for electronic and optoelectronic devices, a deep understanding of optical and electrical parameters and their behavior under various conditions is crucial. Moreover, revealing the underlying physics can lead to new and unforeseen applications of the materials. Both electrical and optical methods have been exploited to assess the charge carrier properties of group-III nitrides and their heterostructures. Commonly, 2DEG mobility and sheet carrier density are obtained from electrical direct-current (DC) Hall effect measurements, which require the fabrication of electrical contacts. Nondestructive and non-invasive measurement of the free charge carriers is key to advance modern materials and device design, but it constitutes a significant challenge. Contactless methods permit the testing of structures without the need for full device processing [9,10,11,12]. The optical Hall effect (OHE) is a contactless method for studying the properties of free and confined charge carriers in semiconductor materials. In addition, it simultaneously provides the dielectric function spectra, which are of high importance for electronic and optoelectronic devices. The significance of the OHE is that it allows obtaining quantitative information about the charge carrier parameters in both bulk materials and heterostructures in a fully contactless manner. In addition, the charge carrier effective mass parameter can be obtained in a wide temperature range, including room temperature and above, along with density and mobility parameters, providing a complete understanding of 2DEG and free carrier transport within the heterostructures. OHE studies of charge carriers in the THz range are often realized employing frequency domain systems employing the continuous-wave THz sources that were widely used to study free charge carrier properties of various electronic materials and device structures [13,14,15,16,17,18]. Studies involving THz time-domain systems [19,20] have also been used, primarily for the measurement of Faraday rotation and Kerr effect [21,22,23].

In this paper, we explore the application of THz OHE to study the charge carrier properties in group-III nitride heterostructures and epitaxial layers. Firstly, we demonstrate the THz OHE results for graded AlGaN channel HEMT structures with different grading profiles and compare their 2DEG properties with the case of conventional (non-graded) AlGaN/GaN HEMT. Secondly, we investigate the anisotropic mobility parameters in a N-polar GaN/AlGaN HEMT structure and discuss the origin of the anisotropy. In the third part, we present a study of the temperature-dependent effective mass parameters of 2DEGs in GaN-based HEMT structures and bulk electrons in GaN epitaxial layers, and review the effective mass parameters determined by OHE reported in the literature.

## 2. Materials and Methods

**Figure 1:**
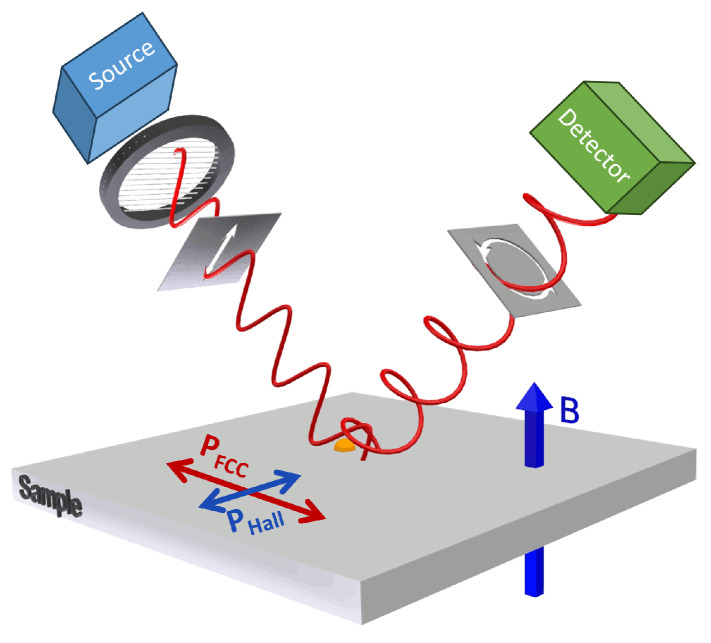
Schematic of the optical Hall effect in a reflection configuration. Free charge carriers produce a material polarization $P_{\mathrm{FCC}}$ following the electric field of an incident electromagnetic waves (analogous to the longitudinal voltage in electrical Hall effect case). The induced polarization $P_{\mathrm{FCC}}$ produces $P_{\mathrm{Hall}}$ due to the Lorentz force, oriented perpendicular to the external magnetic field and the incident electric field vector (analogous to the Hall voltage). $P_{\mathrm{FCC}}$ + $P_{\mathrm{Hall}}$ are the source of the reflected light, which acquires a small circular polarization component due to $P_{\mathrm{Hall}}$, which mainly carries information about the type of the charge carriers, and the cyclotron frequency parameter, while the reflected wave induced by $P_{\mathrm{FCC}}$ is mainly determined by the plasma frequency and broadening parameters. These parameters can be recalculated into carrier density, mobility, and effective mass. If multiple layers are thin enough against the penetration depth of the wave, light interacts with multiple layers and reveals, for example, free carrier properties within buried layers otherwise inaccessible to direct electrical measurements. The figure is adapted from Ref. [11] under CC-BY 4.0 license.

The optical Hall effect is produced by the motion of the free charge carriers under the influence of the external magnetic field as exemplified in Figure 1. An incident electromagnetic wave with linear polarization parallel to the plane of incidence interacting with the conductive sample subjected to an external magnetic field causes displacement of the free charge carriers along the direction of the electric field oscillations. As a result of the Lorentz force, the motion of the free charge carrier deviates from a straight line and adopts a small circular component (Figure 1). The circular component depends on the cyclotron frequency (and as a result on the effective mass parameter), and the Fermi velocity of the free charge carriers. By measuring this change in the polarization state by generalized ellipsometry, access to the free charge carrier sign, the density, mobility, and effective mass, including their anisotropy, is enabled in bulk [11,24,25,26,27] and 2D systems [17,18,28,29,30,31]. In contrast to classical cyclotron resonance and Shubnikov–de Haas oscillations, which require measurements in low-defect density materials to reduce impurity potential scattering and at very low temperatures to reduce phonon scattering, THz OHE provides access to the free charge carrier properties even at room temperature and in materials with a high concentration of defects.

Spectroscopic ellipsometry provides access to the dielectric function tensors of materials by measuring the light polarization change upon reflection (or transmission) from (through) a sample composed of, e.g., a single bulk substrate or multilayered structure [32]. The intrinsic material parameters can be evaluated from the obtained dielectric functions via analysis based on physical models. For example, in the infrared range, phonon excitations contribute to the dielectric function, while in FIR and THz ranges, contributions from free charge carriers become significant for conductive materials. Standard spectroscopic ellipsometry is commonly used to study samples when no conversion between *s*- and *p*-polarization modes (perpendicular and parallel to the plane of incidence, respectively) occurs upon reflection or transmission [32]. In more general cases, when the studied sample is anisotropic and causes conversion between the *s*- and *p*-polarization modes, standard ellipsometry is not sufficient, and generalized ellipsometry must be used, which measures the sample’s optical response in terms of at least 6 frequency-dependent parameters.

Mueller matrix ellipsometry is a powerful technique categorized as generalized ellipsometry. It employs Stokes vector formalism to describe the polarization state of the light beam in terms of 4 real-valued parameters, $S_{0}$, $S_{1}$, $S_{2}$, and $S_{3}$, defined in terms of intensities measured for different light polarization state components [33]:

(1) $$S=\left(\begin{array}{c} S_{0}\\ S_{1}\\ S_{2}\\ S_{3} \end{array}\right)=\left(\begin{array}{c} I_{x}+I_{y}\\ I_{x}-I_{y}\\ I_{+45^{\circ }}-I_{-45^{\circ }}\\ I_{R}-I_{L} \end{array}\right)$$

where $I_{x}$ and $I_{y}$ are intensities of linearly polarized light components along the *x* and *y* directions, which are commonly chosen to match with *s*- and *p*-polarization orientations, respectively ($I_{x}$ = $I_{s}$ and $I_{y}$ = $I_{p}$); $I_{+45^{\circ }}$, $I_{-45^{\circ }}$ are the intensities of light polarized at polarization planes rotated by $45^{\circ }$ clockwise and anti-clockwise, respectively; and $I_{R}$ and $I_{L}$ are the intensities of right-hand and left-hand circular polarization components. Then, the light interaction with the sample is described as the transformation of the incoming beam’s Stokes vector $S_{in}$ into the outgoing beam’s Stokes vector $S_{out}$ by a 4×4 Mueller matrix:

(2) $$S_{out}=\left(\begin{array}{cccc} M_{11} & M_{12} & M_{13} & M_{14}\\ M_{21} & M_{22} & M_{23} & M_{24}\\ M_{31} & M_{32} & M_{33} & M_{34}\\ M_{41} & M_{42} & M_{43} & M_{44} \end{array}\right)S_{in}$$

Typically, the Mueller matrix is normalized by the $M_{11}$ element. The primary advantages of Mueller matrix ellipsometry include obtaining ellipsometry data in real-valued parameters derived from directly measured intensity parameters, and providing information about depolarization effects that may arise during the measurement. Moreover, the formalism grants access to the optical anisotropy of measured samples, which can be readily inferred from the symmetry properties of the Mueller matrix elements.

Measured Mueller matrix data of the THz OHE require model-based data analysis to extract quantitative information about the studied sample. For that reason, an optical model is employed that allows calculation of the Mueller matrix spectra and fitting them to the experimental data by varying model parameters. The optical model is commonly described as a set of layers separated by parallel interfaces representing the sample structure. Each layer is described by its thickness parameter and complex dielectric function tensor $\tilde{\varepsilon }$. The ellipsometric data are calculated using 4×4 transfer matrix formalism [32,34,35]. Fitting is performed iteratively by adjusting the model parameters using a multivariate regression algorithm (the Levenberg–Marquardt method) until the mean-square error function is minimized. This results in the best-match model, which is used to extract the parameters of interest. Error bars are derived from the covariance matrix of the fit parameters simultaneously during the analysis and represent the standard 90% confidence limits. The data analysis presented in this work is performed using the WVase software (version 3) developed by J.A. Woollam Co Inc.

The most technologically relevant crystal structure of group-III nitride materials is the thermodynamically stable wurtzite crystal structure possessing uniaxial optical anisotropy. In such a case, the optical properties can be described by a frequency-dependent dielectric function tensor:

(3) $$\tilde{\varepsilon }=\left[\begin{array}{ccc} \varepsilon_{⊥} & 0 & 0\\ 0 & \varepsilon_{⊥} & 0\\ 0 & 0 & \varepsilon_{\|} \end{array}\right]\;,$$

where $\epsilon_{⊥}$ and $\epsilon_{\|}$ denotes the dielectric function for polarization perpendicular and parallel to the *c*-axis of the wurtzite crystal structure. The dielectric function of group-III nitrides in the THz spectral range is described by the contribution of free charge carriers ${\tilde{\varepsilon }}^{FCC}$, the tail of the phonon contributions in the mid-infrared (MIR) range ${\tilde{\varepsilon }}^{L}$, and an offset related to high-energy electronic excitations ${\tilde{\varepsilon }}_{\infty }$ [11]. It can be written as:

(4) $$\tilde{\varepsilon }={\tilde{\varepsilon }}_{\infty }\left({\tilde{\varepsilon }}^{L}+{\tilde{\varepsilon }}^{FCC}\right)\;.$$

The phonon contribution is described by a factorized four-parameter semi-quantum model containing transverse and longitudinal optical phonon frequencies, ($\omega_{\mathrm{TO}}$, $\omega_{\mathrm{LO}}$) and broadenings ($\gamma_{\mathrm{TO}}$, $\gamma_{\mathrm{LO}}$) as parameters:

(5a) $$\begin{array}{c} {\tilde{\varepsilon }}^{L}=\left(\begin{array}{ccc} \varepsilon_{⊥}^{L} & 0 & 0\\ 0 & \varepsilon_{⊥}^{L} & 0\\ 0 & 0 & \varepsilon_{\|}^{L} \end{array}\right)\;, \end{array}$$

(5b) $$\begin{array}{c} \varepsilon_{j}^{L}=\prod_{i}\frac{\omega_{\mathrm{LO},i,j}^{2}-\omega^{2}-i\omega \gamma_{\mathrm{LO},i,j}}{\omega_{\mathrm{TO},i,j}^{2}-\omega^{2}-i\omega \gamma_{\mathrm{TO},i,j}}\;\left(j=⊥,\|\right)\;, \end{array}$$

with ω being the angular frequency, *j* denoting polarization direction, and *i* indicating different active optical phonon mode pairs. The effect of free charge carriers on the dielectric function is described by the classical Drude model, which is extended to include the contribution of an external static magnetic field and is often referred to as the magneto-optic Drude model. This model contains plasma frequency and broadening parameters, as well as the cyclotron frequency, which depends on carrier density, mobility, and effective mass parameters:

(6) $$\begin{array}{c} {\tilde{\varepsilon }}^{FCC}=-{\tilde{\omega }}_{p}^{2}{\left(\begin{array}{c} \omega^{2}+i\omega \tilde{\gamma_{p}}-i\omega \tilde{\omega_{c}}\left(\begin{array}{ccc} 0 & -b_{z} & b_{y}\\ b_{z} & 0 & -b_{x}\\ -b_{y} & b_{x} & 0 \end{array}\right) \end{array}\right)}^{-1}, \end{array}$$

with screened plasma frequency tensor ${\tilde{\omega }}_{p}$, effective mass tensor ${\tilde{m}}^{*}$, plasma broadening tensor ${\tilde{\gamma }}_{p}$, and cyclotron frequency tensor ${\tilde{\omega }}_{c}$ defined as:

(7) $${\tilde{\omega }}_{p}^{2}=\frac{Nq^{2}}{\varepsilon_{0}}{\tilde{\varepsilon }}_{\infty }^{-1}{\tilde{m}}^{*-1},\;{\tilde{m}}^{*}=\left[\begin{array}{ccc} m_{x}^{*} & 0 & 0\\ 0 & m_{y}^{*} & 0\\ 0 & 0 & m_{z}^{*} \end{array}\right],\;{\tilde{\gamma }}_{p}=\left[\begin{array}{ccc} \tau_{x}^{-1} & 0 & 0\\ 0 & \tau_{y}^{-1} & 0\\ 0 & 0 & \tau_{z}^{-1} \end{array}\right],\;{\tilde{\omega }}_{c}=q\left|\vec{B}\right|{\tilde{m}}^{*-1}\;.$$

Here, *N*, *q*, $\varepsilon_{0}$ are the carrier concentration, charge carrier unit charge, and vacuum permittivity parameter, while τ is the scattering time. The mobility parameter can be obtained from the scattering time and effective mass parameters using the relation $\tilde{\mu }=q\tilde{\tau }{\tilde{m}}^{*-1}$. The magnetic field vector is defined as $\vec{B}=\left|\vec{B}\right|\left(b_{x},b_{y},b_{z}\right)$. Normally, the effective mass and scattering time parameters are isotropic for polarization orthogonal to the c-axis; therefore, they can be written as $m_{⊥}^{*}=m_{x}^{*}=m_{y}^{*}$ and $\tau_{⊥}=\tau_{x}=\tau_{y}$, while $m_{\|}^{*}=m_{z}^{*}$ and $\tau_{\|}=\tau_{z}$. In case of lack of detectable anisotropy, all the effective mass and scattering time parameters can be replaced with the two scalar parameters $m^{*}$ and τ. Regardless of whether the intrinsic anisotropy of the charge carrier properties is present or not, the free charge carrier contribution to the dielectric function tensor, ${\tilde{\varepsilon }}^{FCC}$, gives rise to non-zero off-diagonal block Mueller matrix elements ($M_{ij}$, ij=13,14,23,24,31,32,41,42) whenever the external magnetic field is present, resulting in optical birefringence that we refer to as the OHE.

The OHE measurements were performed using a THz frequency domain ellipsometer that was designed and built using a stealth technology approach to reduce the formation of standing waves. The system is described in detail in Reference [36] and covers a spectral range from 100 GHz to 1 THz. The THz ellipsometer operates in a polarizer-sample-rotating analyzer arrangement. In the Mueller matrix ellipsometry mode, it is capable of measuring the 3×3 upper left block ($M_{ij}$/$M_{11}$, where i,j=1,2,3) of the 4×4 Mueller matrix ($M_{ij}$/$M_{11}$, where i,j=1,2,3,4). The THz system has a continuous wave solid-state source with a base frequency from 100 GHz to 170 GHz, which is augmented by Schottky-diode frequency multipliers, ×2, ×3, and ×6 (Virgin Diodes, Charlottesville, VA, USA), providing the accessible range of 100–1000 GHz with a few narrow gaps that are not covered. The ellipsometer can perform cavity-enhanced OHE measurements at room temperature in reflection geometry using a permanent neodymium magnet (0.6 T) [37]. In addition, the system is augmented by a superconducting magnet with a cryostat (Cryogenics Ltd., London, UK), which allows performing OHE measurements in reflection or transmission modes at magnetic fields up to 8 T and sample temperatures from 2 K to 400 K. Conductive layers with thickness parameters much smaller than the wavelength of THz radiation (in our case ranging from 3 mm to 300 μm in the frequency range of 100 GHz–1 THz) behave as 2D conductive channels. Therefore, only the sheet carrier density ($N_{s}=Nd$, where *N* is carrier concentration and *d* is layer thickness) can be obtained from the THz OHE data analysis. To obtain the bulk carrier concentration, the thickness parameters of conductive layers must be measured independently and fixed in the model analysis. For the studies presented in this paper, the thickness parameters of thin layers were measured independently using Vis-UV ellipsometry (RC2, J.A. Woollam Co. Inc., Lincoln, NE, USA).

The studied group-III nitride epitaxial layers and device heterostructures were grown on semi-insulating 4H-SiC substrates using a hot-wall metal-organic chemical vapor deposition (MOCVD) reactor [38,39,40]. The standard characterization of the grown layers included X-ray diffraction (XRD) for compositional and crystal quality studies and electrical sheet resistance, capacitance–voltage (C-V) and Lehighton mobility measurements for carrier density and mobility parameters of 2DEG in HEMTs, and electrons in doped layers. For selected samples, the Al-content profiles and the structural quality were determined by scanning transmission electron microscopy (STEM) combined with energy-dispersive X-ray spectroscopy (EDS) [40,41]. The measurements were performed using the double corrected Linköping FEI ${\mathrm{Titan}}^{3}$ 60–300 microscope (Linköping, Sweden), operated at 300 kV. The built-in Super-X/Quantax EDS system (Bruker, Billerica, MA, USA) was employed, and the absolute quantification was made using the Esprit software (https://www.espritcam.com/, accessed on 29 December 2023) and its built-in calibrations for TEM-EDS.

## 3. Results

### 3.1. Graded AlGaN Channel HEMTs

**Figure 2:**
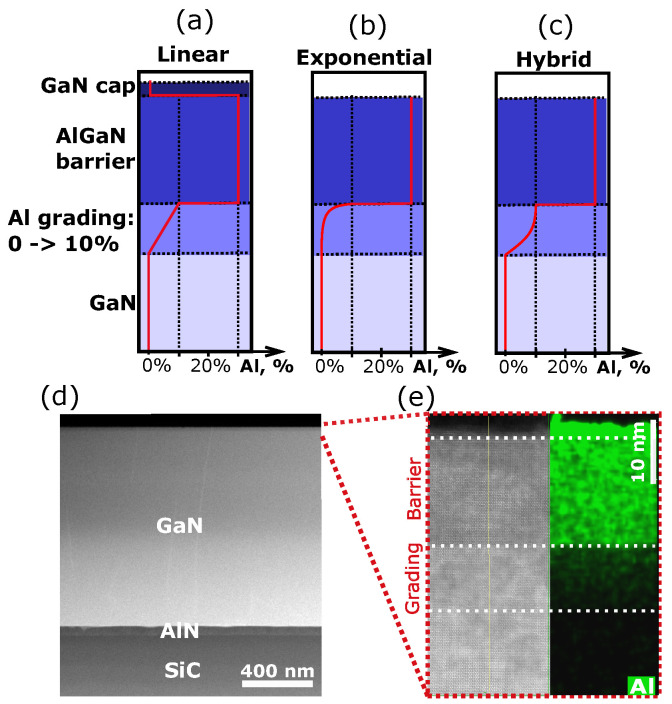
Graded channel AlGaN/GaN HEMT structures: (**a**–**c**) schematic drawings of the heterostructures with the nominal Al grading profiles in the channel, (**d**) STEM image of the linearly graded HEMT structure and (**e**) the EDS map of Al distribution in the vicinity of the AlGaN/GaN interface.

Incorporating a thin AlGaN layer with Al composition grading as a channel at the interface between the GaN buffer and AlGaN barrier layers in AlGaN/GaN HEMTs modifies the distribution of the 2DEG carriers formed at the interface. The graded layer induces delocalization of the electrons’ wavefunction which is now allowed to extend across the graded layer [40,42]. Here, we refer to the 2DEG channel formed at such graded channel HEMTs as a three-dimensional electron gas (3DEG) channel. Such spatially broader distribution in the conductive channel can improve the HEMT device linearity at high powers [40,42,43,44,45]. Here, we present THz OHE results for graded channel AlGaN/GaN HEMT structures with different grading profiles [40]. The HEMT structures consist of the following stack of layers on semi-insulating 4H-SiC substrate: AlN (60 nm)/GaN buffer (1100 nm)/graded ${\mathrm{Al}}_{x}$G$a_{1-x}$N channel (*x* = 0–0.1, 10 nm)/A$l_{x}$G$a_{1-x}$N barrier (x=0.3). Three different grading profiles were implemented: exponential, linear, and hybrid (hyperbolic tangent-linear). The growth conditions can be found in Ref. [40]. The studied structures with nominal Al grading profiles of the channel layer are shown in Figure 2a–c. The linearly graded channel HEMT also contains a GaN cap (2–3 nm) layer on top of the structure. The intended Al grading in the channel was confirmed by STEM and EDS measurements. Figure 2d,e show a representative STEM image and the respective EDS map of the Al content. Due to the very low thickness, it is difficult to resolve the GaN cap layer in the STEM image and the respective EDS map in Figure 2e. The high Al content layer that appears on top of the structure is an artifact related to the TEM sample preparation and is associated with the Al oxide that can form on top of the structure. The surface roughness (RMS) of the HEMT structures determined from 5 × 5 μm atomic force microscopy (AFM) images are 0.32 nm, 0.45 nm, and 0.28 nm for the exponential, hybrid, and linear grading channel HEMTs, respectively.

**Figure 3:**
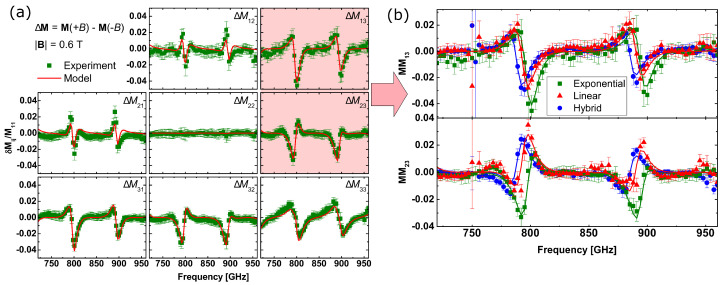
(**a**) The experimental (squares) and the best-match model (solid line) Mueller matrix difference spectra (Δ**M**(±0.6T) = **M**(+0.6T) −**M**(−0.6T)) for the exponentially graded channel AlGaN/GaN-HEMT. (**b**) Comparison of the selected experimental (squares, triangles, circles) and best-match model (solid lines) off-diagonal-block Mueller matrix element spectra (Δ$M_{13,23}$) for the graded channel AlGaN/GaN HEMTs with different grading profile in the channel.

The THz OHE measurements were performed at room temperature in reflection geometry using the permanent neodymium magnet (0.6 T) and employing a backside cavity for OHE enhancement [36,37]. The measurements were carried out on both sides of the magnet, resulting in Mueller matrix datasets obtained at two opposing magnetic field directions (**M**[±0.6T]). The experimental difference Mueller matrix spectra (Δ**M**[±0.6T] = **M**[+0.6T] −**M**[−0.6T]) and the best-match model spectra for the exponentially graded channel HEMT are shown in Figure 3a, and the spectra for the selected off-diagonal-block Mueller matrix elements (Δ$M_{13,23}$) are shown for all HEMT structures in Figure 3b. Specifically, the off-diagonal block elements are proportional to the cyclotron frequency $\omega_{c}$ and provide sensitivity for this parameter. The experimental difference Mueller matrix spectra reveal OHE signatures following the Fabry–Perot oscillations pattern within the sample–air gap–mirror optical system [13,37], with the strongest OHE signatures in the spectral ranges where the reflectivity is the smallest. The lineshapes and amplitudes of these features are governed by the free electron properties. More specifically, the features observed in the Mueller matrix difference spectra are caused by differences between left- and right-hand circularly polarized light propagation due to the magneto-optic anisotropy of the free charge carriers. Therefore, the differences between the Mueller matrix spectra among the samples indicate different free-charge carrier properties of the 3DEG channel. In addition, the spectral position of the observed features is also affected by the substrate and cavity thickness parameters. Note that the Mueller matrix difference spectra are equal to zero in cases where there are no free-charge carriers in the structure. The 3DEG parameters extracted from the best-match model analysis are shown in Table 1.

The sensitivity of the THz OHE to the out-of-plane anisotropy, i.e., to the parameters $\tau_{\|}$ and $m_{\|}^{*}$, is very low for such thin conductivity channels, therefore these parameters are always assumed to be equal to the in-plane parameters. Here, the model used for data analysis further assumes the effective mass and mobility parameters being in-plane isotropic. The very good agreement between the experimental data and the model shows that no detectable in-plane anisotropy of the 3DEG properties for the studied structures can be inferred.

For all graded HEMT structures, the obtained sheet density parameters are in the range of $N_{s}=1.1\times 10^{13}-1.3\times 10^{13}$ ${\mathrm{cm}}^{-2}$ and can be considered to be the same within the 90% confidence limits. Mobility values for all the samples are significantly lower than those in conventional (non-graded channel) AlGaN/GaN HEMT structures with sharp interfaces and typical mobilities around 2000 ${\mathrm{cm}}^{2}$$V^{-1}$$s^{-1}$ and above [18,40]. This can be anticipated since the alloy scattering in the graded channel is expected to be significantly stronger as compared to the case of non-graded conventional HEMTs. We find that the exponentially graded channel-HEMT has slightly higher mobility (∼30%), $\mu^{exp}=960\left(\pm 60\right)$ ${\mathrm{cm}}^{2}$$V^{-1}$$s^{-1}$, in comparison with the hybrid and linear grading structures with mobility values of $\mu^{hybrid}=730\left(\pm 50\right)$ ${\mathrm{cm}}^{2}$$V^{-1}$$s^{-1}$ and $\mu^{linear}=720\left(\pm 40\right)$ ${\mathrm{cm}}^{2}$$V^{-1}$$s^{-1}$, respectively. This could be explained by the effectively sharper potential barrier for the 3DEG and as a result of a lower Al concentration in the channel region where most of the electrons are localized, resulting in a lower alloy scattering. The exponential grading channel HEMT structure has the lowest 3DEG effective mass parameter. That further supports the statement that electrons in the exponentially graded channel are more localized in the region with lower Al concentration. Note that this leads to a lower effective mass since the latter increases with Al content [25]. As the mobility parameter inversely depends on the effective mass ($\mu =e\tau m^{*-1}$), the lower effective mass partly explains the higher mobility for this sample. However, the difference in effective masses is only around 10%. Therefore, this cannot fully account for the higher 3DEG mobility parameter in the exponential grading channel, showing that a longer scattering time is associated with the HEMT. We note that the results obtained from the THz OHE on mobility parameters and sheet density are in good agreement with the results from C-V, Lehighton mobility, and Eddy current measurements [46]. More information about the graded-channel AlGaN/GaN HEMT structures, their structural and electrical properties, and the respective device performance can be found in Ref. [40].

### 3.2. Anisotropic Mobility in N-Polar GaN/AlGaN HEMT

Nitrogen-polar III-nitride heterostructures offer advantages over metal-polar structures in high-frequency and high-power applications [47]. Due to the inverted polarization field in N-polar structures as compared to their Ga-polar counterparts, the 2DEG channel forms at the interface between the top GaN layer and the AlGaN back barrier. This enables enhanced 2DEG confinement and reduction in the on-resistance, resulting in improved power efficiency. The N-polar GaN/AlGaN HEMT structure studied here was grown on 4H-SiC (000$\bar{1}$) substrate with miscut angle of 4° toward the m-plane (10$\bar{1}$0). The high-temperature AlN nucleation layer was grown on that substrate, which was followed by the growth of the GaN buffer layer using a multi-step temperature process [48,49], the ${\mathrm{Al}}_{0.31}$G$a_{0.69}$N back barrier and the GaN channel layer on top. The surface roughness (RMS) determined from 5 × 5 μm AFM images was 2 nm.

**Figure 4:**
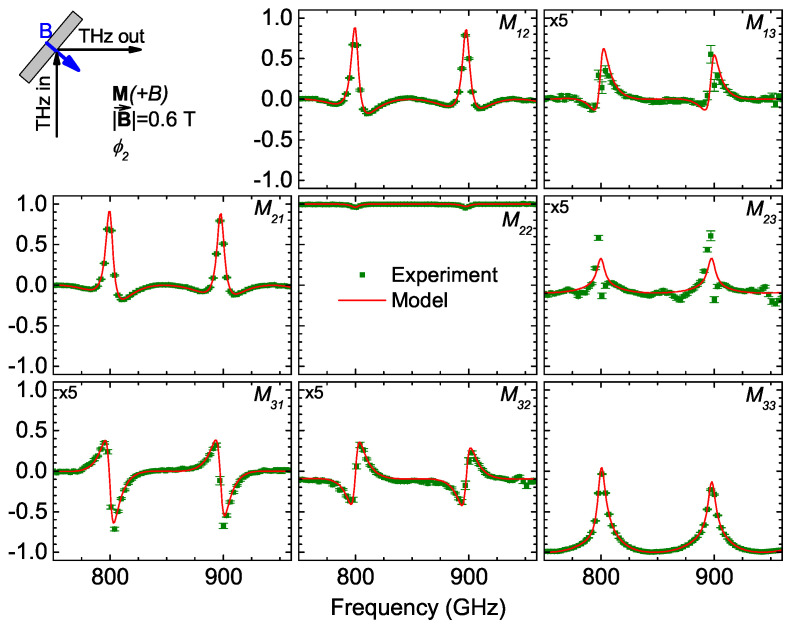
THz-OHE Mueller matrix spectra (**M**[+0.6T]) for N-polar GaN/AlGaN HEMT structure measured in reflection geometry (at angle of incidence of 45°) with the sample mounted on the permanent neodymium magnet and implemented backside cavity.

The THz OHE measurements were performed in reflection geometry (angle of incidence 45°) using the permanent neodymium magnet (0.6 T) and employing a backside cavity [37]. The measurements were taken at both sides of the neodymium magnet, resulting in two opposing magnetic field directions (**M**[±0.6T]). The Mueller matrix spectra for positive magnetic field (**M**[+0.6T]) are shown in Figure 4. Most of the Mueller matrix spectra contain distinct spectral features modulated by the Fabry–Perot oscillations within the mirror(magnet)–cavity–sample optical system. The red solid lines indicate the best-match model data, which can be seen to be in perfect agreement with the experimental data points.

**Figure 5:**
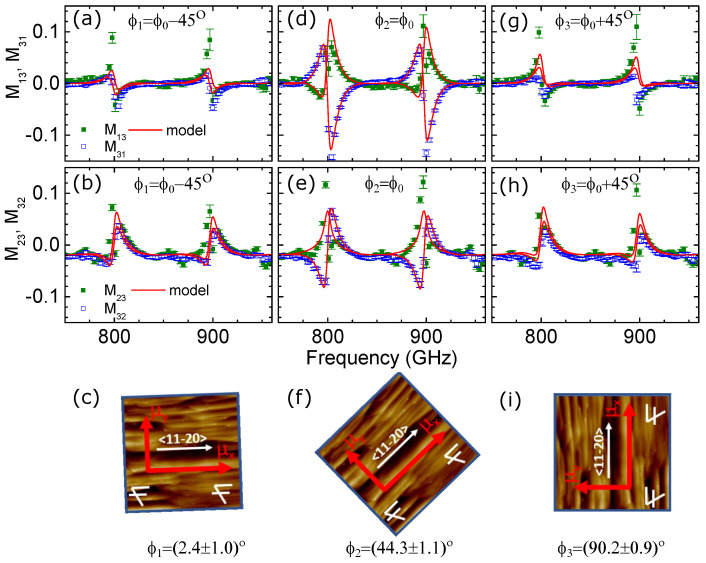
Experimental (symbols) and the best-match model (solid lines) THz-OHE off-diagonal block Mueller matrix elements spectra ($M_{13}$, $M_{23}$, $M_{31}$, $M_{32}$) for N-polar GaN/AlGaN HEMT structure measured at 3 different azimuth orientations (**a**,**b**—$\phi_{1}$, **d**,**e**—$\phi_{2}$, and **g**,**h**—$\phi_{3}$). The azimuth angles obtained from the best-match optical model representing the angle between the fast mobility axis $\mu_{x}$ and the plane of incidence, and the AFM images showing the absolute sample orientation for each measurement are depicted in (**c**,**f**,**i**).

The THz OHE measurements revealed a significant dependence of the Mueller matrix spectra on the azimuth orientation (in-plane rotation of the sample with respect to the surface normal) of the N-polar HEMT sample. The measured Mueller matrix spectra of the block-off diagonal elements ($M_{13,23,31,32}$) for three different azimuth orientations ($\phi_{1}$, $\phi_{2}$, $\phi_{3}$) are shown in Figure 5. Each column represents the different azimuth orientation with a nominal 45° rotation in between. Such azimuth dependence of the Mueller matrix spectra is a direct fingerprint of the in-plane optical anisotropy of the sample. Without the presence of in-plane anisotropy, the OHE induces symmetric off-diagonal block Mueller matrix elements, i.e., $M_{ij}=M_{ij}$, with ij=13,23,31,32. Any in-plane anisotropy causes antisymmetric Mueller matrix spectra $M_{ij}=-M_{ij}$, with ij=13,23,31,32 at B = 0 T, whenever the anisotropy axis is not parallel to the plane of incidence. In Figure 5a,b,d,e,g,h, one can observe that different azimuth angles result in different symmetry properties of the off-diagonal block Mueller matrix elements. At the azimuth angles $\phi_{1}$ and $\phi_{3}$, the spectra are nearly symmetric ($M_{ij}\approx M_{ji}$); however, the spectra are not the same for the two different orientations. Meanwhile, the spectra at $\phi_{2}$ show more complicated asymmetric behavior as a result of the convolution of the magneto-optical and in-plane anisotropy effects, which correspond to symmetric and antisymmetric parts.

Our model-based data analysis revealed that the observed anisotropy is induced by the directionally dependent mobility parameter of the 2DEG channel. Two different in-plane mobility parameters ($\mu_{x}$ and $\mu_{y}$) for the 2DEG in the magneto-optic Drude model had to be implemented in the model in order to fit the experimental data with the model. The out-of-plane mobility parameter $\mu_{z}$ was set to zero due to the limited sensitivity to the out-of-plane optical properties for such a thin layer (2DEG is localized within a few nanometers). Therefore, in the best-match model, the dielectric function for the 2DEG channel contains the Drude contribution, with the mobility parameter expressed as a second-rank tensor with the on-diagonal elements representing the mobilities in the three different orthogonal directions being different. The azimuth angle ϕ indicates the angle between the plane of incidence and the $\mu_{x}$ mobility axis. It is a fitting parameter in the model analysis and therefore was extracted from the best-match model. When mobility axes are nearly aligned with the plane of incidence (at $\phi_{1}=2.4^{\circ }$ and $\phi_{3}=90.2^{\circ }$), the Mueller matrix spectra are nearly symmetric and therefore, the spectral features in Figure 5 are mainly induced by the OHE. At the azimuth angle $\phi_{2}=44.3^{\circ }$, the $\mu_{x}$ mobility axis makes an angle with the plane of incidence, and therefore the Mueller matrix spectra (in Figure 5d,e) reflect the combined magneto-optic and in-plane anisotropy-induced effects.

The in-plane mobility parameters extracted from the best-match model are $\mu_{x}=1170\pm 20$ and $\mu_{y}=820\pm 10$. The higher mobility parameter along the fast axis $\mu_{x}$ is around 40% larger than the mobility along the slow axis. This results in significant anisotropy as observed in the Mueller matrix spectra variation with the azimuth orientation of the sample (Figure 5). The determined sheet carrier density was $N_{s}=7.8\left(\pm 0.3\right)\times 10^{12}$ ${\mathrm{cm}}^{-2}$, while the extracted effective mass parameter was 0.258(±0.002) $m_{0}$.

Additional AFM measurements of the N-polar HEMT structure were performed in order to correlate the THz OHE results with the surface morphology (see Figure 5c,f,i). The absolute azimuth orientation of the sample in the AFM measurements was recorded and correlated with the azimuth angles of the fast and slow mobility axis extracted from the THz OHE measurements. The AFM shows a step-like surface morphology, typical for the growth on the off-axis SiC substrate [48]. The AFM results further reveal that the mobility axes follow the orientation of the steps with the fast mobility axis $\mu_{x}$ being oriented along the steps parallel to the [11$\bar{2}$0], while the slow mobility axis $\mu_{y}$ is oriented across the steps i.e., along the [1$\bar{1}$00] (see Figure 5c,f,i). This suggests that the step-like morphology also translates into the GaN/AlGaN interface where 2DEG is located and introduces the anisotropic mobility. The results indicate that steps introduce additional scattering of the 2DEG for electrons moving in the direction perpendicular to the steps. A similar effect is observed for another 2D electronic system on SiC substrate, namely, epitaxial graphene , where the step-like surface morphology of the substrate introduces additional carrier scattering, leading to significant in-plane optical anisotropy observed by OHE measurements [17].

### 3.3. Temperature Dependence of Electronic Properties

The OHE measurements allow accessing the charge carrier properties in a wide temperature range, which is mainly limited by the capabilities of the current experimental setup (2 K to 400 K). A major advantage of OHE is the access to the effective mass parameter at elevated temperatures along with the free charge carrier density and mobility (or scattering time). The OHE data are sensitive to the cyclotron frequency parameter which depends on the effective mass even when the former is outside the measured frequency range. This allows determination of the effective mass parameter from model-based analysis of the OHE data. Commonly used methods for effective mass determination, such as Shubnikov–de Haas oscillations or cyclotron resonance measurements, require high mobilities, low temperatures, and high magnetic fields. In contrast, OHE has been demonstrated to provide effective mass parameters even at room temperature or above for various electronic materials at magnetic fields as low as 0.5 T [13,18,24,25,26,27,28,36,37,50].

**Figure 6:**
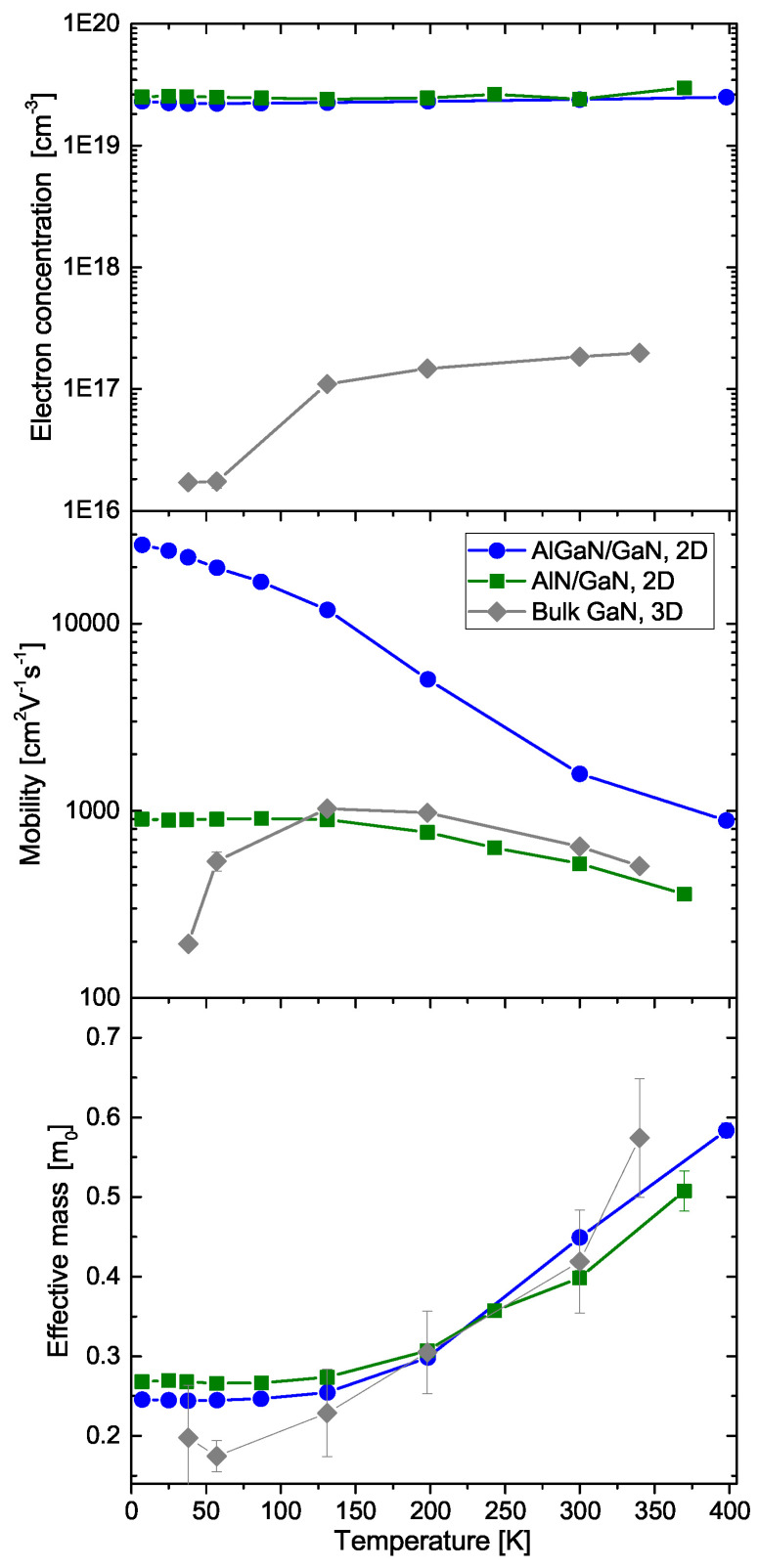
Temperature dependence of electron concentration, mobility and effective mass parameters of 2DEG in AlGaN/GaN (nominal Al content of 29%) and AlN/GaN HEMT structures, and bulk carriers (denoted as 3D) in GaN substrate doped with Si (nominal Si concentration $N_{\mathrm{Si}}=1\times 10^{17}$ ${\mathrm{cm}}^{-3}$) [31]. The electron concentration for the AlGaN/GaN and AlN/GaN HEMTs structures is calculated from the sheet density parameters $N_{s}$, obtained from the THz OHE analysis, and assuming a 4 nm layer thickness and uniform distribution across the channel ($N=N_{s}/d$).

Bulk free charge carriers in group-III nitrides are generated by a donor, i.e., Si, and an acceptor, i.e., Mg doping. On the other hand, AlGaN/GaN HEMT structures contain 2DEG channel at the interface between two nominally undoped layers with different bandgaps, and different spontaneous and piezoelectric polarizations [51,52]. Bulk electrons and 2DEG have very different electrical properties, which stipulate their different temperature dependencies. The 2DEG channel demonstrates much higher mobility values in comparison to the bulk carriers. Two HEMT structures containing 2DEG and an n-type doped GaN substrate containing bulk (3D) free electrons were selected for temperature dependence study. The HEMT structures were grown epitaxially on semi-insulating 4H-SiC (0001) substrates using an AlN nucleation layer with a nominal thickness of 60 nm followed by a nominally 1.9 um-thick relaxed GaN buffer layer, and by a 10–20 nm thick pseudomorphic AlGaN (nominal Al content of 29%) or AlN barrier layers [40]. The n-type GaN substrate (MSE Supplies) contains a nominal Si concentration of $N_{\mathrm{Si}}=1\times 10^{17}$ ${\mathrm{cm}}^{-3}$ [31]. The THz OHE measurements of the samples were performed in the spectral range of 700–1000 GHz. The charge carrier properties of bulk 3D electrons in the GaN substrate, and 2DEGs in the AlGaN/GaN and AlN/GaN HEMT structures obtained from THz OHE in a wide temperature range from 4.5 K to 400 K are shown in Figure 6.

For comparative purposes, in Figure 6, the electron concentrations for AlGaN/GaN and AlN/GaN HEMTs are calculated from the obtained sheet density parameters $N_{s}$, assuming a 4 nm layer thickness and uniform distribution across the channel ($N=N_{s}/d$, where d=4 nm). As expected, the 2DEG density remains virtually unchanged with temperature, while the carrier density of the n-type doped GaN substrate gradually increases with temperature due to the thermal activation of the donor states. The mobility behavior is also markedly different. For the 2DEG, the mobility decreases with increasing temperature. In contrast, for n-type GaN, the electron mobility parameter first increases, reaching a maximum value of around 150–200 K, after which it decreases with increasing temperature. For both bulk carriers and 2DEG, the mobility at high temperatures is limited by optical phonon scattering, while at low temperatures, the scattering mechanisms limiting the mobility are different [53,54,55]. For bulk (3D) carriers, the low-temperature mobility is limited by impurity scattering, which is negligible for the 2DEG [53,54,56]. For the 2DEG, the low-temperature mobility is limited by either alloy disorder or interface roughness scattering. The AlN/GaN HEMT has significantly lower mobility compared to the AlGaN/GaN HEMT, which could be related to higher alloy disorder scattering. We observed in similar structures grown by MOCVD that achieving a pure AlN barrier layer is very difficult. Instead, it commonly results in a compositionally graded Al-rich AlGaN layer, causing strong delocalization of the electron wavefunction and high penetration into the barrier layer (results to be published elsewhere).

Despite the differences in the temperature dependencies of the bulk and 2DEG carrier density and mobility parameters, the results for the respective effective mass parameters are very similar. For all of the studied samples, at low temperatures, the effective mass parameter value is close to the well-established literature value of 0.23$m_{0}$ [57,58], while above 150 K, it shows an increase with temperature. Initially, such a temperature dependence of the effective mass parameter was observed for 2DEG in AlGaN/GaN HEMT structures without an AlN inter-layer between the buffer and the barrier layers and assigned to the 2DEG wavefunction hybridization due to a penetration into the AlGaN barrier layer [18,28]. However, such a strong increase with temperature cannot be explained by hybridization alone, nor can it be explained by conduction band non-parabolicity. Polaronic effects due to the coupling of electrons to the optical phonons could also be excluded as a sole reason for the effective mass enhancement, as they only account for about 10 % increase. A detailed study of the electron effective mass parameter increase at high temperatures in bulk GaN was recently reported in Ref. [31]. It was suggested that possible deviation of the free charge carrier behavior from the classical Drude model commonly used to describe conductivity in group-III nitrides (leading to the renormalization of the effective mass parameter) may play an important role for the observed increase in the electron effective mass parameter at high temperatures and THz frequencies [31].

**Figure 7:**
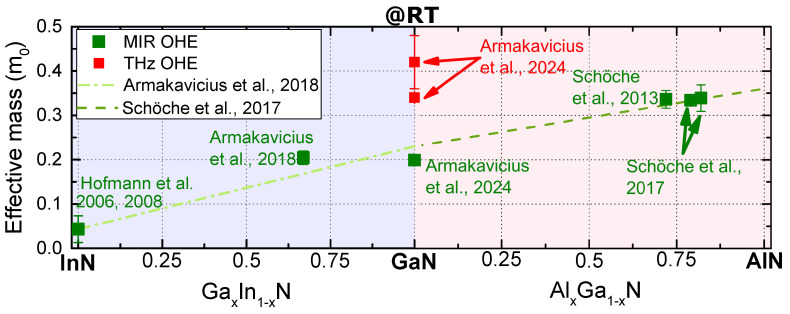
Summary of the published bulk (3D) electron effective mass parameters (isotropically averaged) at room temperature (RT) in group-III nitrides determined from MIR OHE [24,25,26,50,59]. The bulk effective mass of GaN obtained from THz-OHE at RT for n-type bulk GaN substrate and epitaxial layer on SiC are also included for comparison [31]. The dashed lines indicate linear dependencies of the electron effective mass parameters for ${\mathrm{Ga}}_{x}$${\mathrm{In}}_{1-x}N$ (conduction band minima) and ${\mathrm{Al}}_{x}$${\mathrm{Ga}}_{1-x}N$ published in references [25,26] reporting MIR OHE results.

Table 2 summarizes the published effective mass parameters obtained via Drude model-based analysis of the THz OHE measurements of various group-III nitride heterostructures containing 2DEG conductivity channels. The RT electron effective mass parameter of 2DEG is close to 0.30 $m_{0}$ for HEMTs with a GaN channel. This value is higher than the well-accepted value of 0.23 $m_{0}$ for bulk GaN. The latter, however, is primarily determined by infrared spectroscopy, cyclotron resonance or Shubnikov–de Haas oscillations techniques at low temperatures. The 2DEG effective mass parameter of high Al content AlGaN channel HEMT is even higher than $0.60\;m_{0}$. Figure 7 summarizes the bulk effective mass parameters obtained from MIR OHE for group-III nitride materials [24,25,26,50,59,60,61,62,63,64]. The bulk effective mass parameter for GaN obtained from THz-OHE is included for comparison and can be seen to be significantly higher [31].

### 3.4. Conclusions

In summary, we present the application of THz OHE to study charge carrier properties of group-III nitride heterostructures and bulk material using several examples of contemporary research significance. We show that in graded channel AlGaN/GaN HEMT structures for linear power amplifiers and receivers, exponential grading of the Al content over 10 nm of the channel thickness leads to higher mobility and a lower effective mass parameter as compared to the cases of linear and hybrid Al grading profiles. The results suggest that exponential grading causes free electrons to be localized in the channel region with lower Al content, resulting in reduced alloy scattering. THz OHE study of an N-polar GaN/AlGaN HEMT structure grown on a SiC substrate with off-cut angle toward the m-plane reveals strong optical anisotropy inferred from the symmetry properties of the measured Mueller matrix spectra. Data analysis reveals in-plane anisotropic electron mobility due to directionally dependent 2DEG scattering induced by the step-like surface morphology, with higher mobility along the steps parallel to the [$11\bar{2}0$] plane. We also compare free-charge carrier properties over a wide temperature range from 4.5 K to 400 K between 2DEG and bulk electrons in GaN. An increase in the electron effective mass parameter with the temperature is observed in both cases independently of the differences between 2D and bulk carriers. This observation is consistent with the prevalence of the reported GaN electron effective mass parameters in the literature. Such behavior could be potentially explained by a deviation of the charge carrier behavior from the Drude model, commonly used to describe conductivity in group-III nitride materials, in addition to the non-porabolicity, polaronic, and hybridization effects. These studies showcase THz-OHE as a powerful tool for studying the free and confined charge carrier properties of group-III nitride materials and heterostructures at different temperatures, with high sensitivity to their anisotropy.

Exciting new opportunities are presented by the future development of OHE measurements of group-III-nitride device structures under different conditions relevant to the device operation. Further development of the THz OHE technique can enable in situ studies of the charge carrier properties under electric gating, and high temperatures in fully contactless manner, which can further advance our understanding of the factors affecting device performance and can provide routes to improve it beyond the current state of the art.

## Figures and Tables

**Table 1:** The 3DEG parameters of the graded-channel AlGaN/GaN HEMTs with different grading profiles obtained from the THz OHE best-match model.

Channel Grading	Sheet Density, $10^{13}$ ${\mathrm{cm}}^{-2}$	Mobility, ${\mathrm{cm}}^{2}$$V^{-1}$$s^{-1}$	Effective Mass, $m_{0}$
Exponential	1.05±0.18	960±60	0.40±0.03
Hybrid	1.11±0.09	730±50	0.44±0.03
Linear	1.27±0.15	720±40	0.43±0.02

**Table 2:** Summary of the effective mass parameters for the 2DEG in HEMT structures obtained from THz-OHE.

Structure	Substrate	Temp., K	Mag. Field, T	Exp. Eff. Mass, $m_{0}$	Ref.
1) ${\mathrm{Al}}_{0.82}$${\mathrm{In}}_{0.18}$N/AlN/GaN	${\mathrm{Al}}_{2}$ $O_{3}$	RT	0.55	0.27(±0.01)	[13]
2) ${\mathrm{Al}}_{0.75}$${\mathrm{Ga}}_{0.25}$N/GaN	4H-SiC	RT	0.55	0.32(±0.01)	[36]
3) ${\mathrm{Al}}_{0.19}$${\mathrm{Ga}}_{0.81}$N/GaN	4H-SiC	RT	0.55	0.30(±0.01)–0.32(±0.01)	[18]
4) ${\mathrm{Al}}_{0.82}$${\mathrm{In}}_{0.18}$N/AlN/GaN	${\mathrm{Al}}_{2}$ $O_{3}$	RT	0.55	0.24(±0.02)	[37]
5) ${\mathrm{Al}}_{0.25}$${\mathrm{Ga}}_{0.75}$N/GaN	4H-SiC	1.5–300	3	0.22(±0.01)–0.36(±0.03)	[28]
6) * AlN/${\mathrm{Al}}_{0.78}$${\mathrm{Ga}}_{0.22}$N	4H-SiC	5	8	0.63(±0.04)	[30]
7) ${\mathrm{Al}}_{0.29}$${\mathrm{Ga}}_{0.71}$N/GaN	4H-SiC	7–400	8	0.25(±0.01)–0.58(±0.01)	This work
8) AlN/GaN	4H-SiC	7–370	8	0.27(±0.01)–0.51(±0.03)	This work

* Note that this structure contains 2DEG in ${\mathrm{Al}}_{0.78}$${\mathrm{Ga}}_{0.22}$N, while all other HEMTs contain 2DEG in GaN.

## Data Availability

The data presented in this study are available on request from the corresponding author. The data are not publicly available due to privacy or ethical restrictions.

## Abbreviations

The following abbreviations are used in this manuscript:

THz	Terahertz
GHz	Gigahertz
OHE	Optical Hall Effect
HEMT	High-electron-mobility transistor
2D	Two-dimensional
3D	Three-dimensional
2DEG	Two-dimensional electron gas
3DEG	Three-dimensional electron gas
RT	Room temperature
GaN	Gallium nitride
AlN	Aluminum nitride
AlGaN	Aluminum gallium nitride
AlINN	Aluminum indium nitride
SiC	Silicon carbide
Vis	Visible
UV	Ultraviolet
MOCVD	Metal-organic chemical vapor deposition
XRD	X-ray diffraction
